# Breaking the Mold: New Strategies for Fighting Aflatoxins

**DOI:** 10.1289/ehp.121-a270

**Published:** 2013-09-01

**Authors:** Charles W. Schmidt

**Affiliations:** **Charles W. Schmidt**, MS, an award-winning science writer from Portland, ME, has written for *Discover Magazine*, *Science*, and *Nature Medicine*.

In 2010 Kenyan authorities reported that 2.3 million bags of corn harvested in that country had been contaminated with fungal poisons known as aflatoxins.^^1^^ These toxins—which include aflatoxin B_1_, the most potent naturally occurring liver carcinogen ever identified—are produced by *Aspergillus flavus* and *A. parasiticus*, and they infect corn (maize), nuts, and other crops, especially during periods of drought stress and intense heat. Aflatoxins have been implicated in poisoning outbreaks that killed hundreds of people in developing countries, and experts suspect many aflatoxin-related fatalities go unreported.^^2^^

Toxicity risks from aflatoxins are very low in the United States and other developed countries, according to Charles Hurburgh, a professor of agricultural engineering and an extension grain specialist at Iowa State University. People in these countries eat a wide variety of foods with little or no risk of aflatoxin contamination, and for those foods where aflatoxins may occur, contamination is closely monitored and tightly regulated.

However, chronic exposures are endemic in developing countries, because aflatoxin monitoring is inadequate, populations tend to rely heavily on just a few staple crops that are vulnerable to *Aspergillus* infection, and growing conditions often favor mold growth. In a recent analysis of data from the National Health and Nutrition Examination Survey, only 1.3% of more than 2,000 U.S. blood samples had detectible levels of aflatoxins,^^3^^ compared with 78% of more than 3,000 blood serum samples from the nationally representative Kenya AIDS Indicator Survey.^^4^^

The U.S. Centers for Disease Control and Prevention estimates that 4.5 billion people in the developing world may have chronic exposure to aflatoxins in the diet.^^5^^ And according to one analysis, these exposures account for between 25,200 and 155,000 cases of liver cancer every year, particularly in Asia and sub-Saharan Africa.^^6^^ Now aflatoxins are drawing international attention from development groups and aid agencies, who are teaching farmers and buyers how to spot and combat the pervasive threat.

## Aflatoxins Under the Spotlight

Several developments account for the increased focus. First, there is mounting evidence that aflatoxins cause or exacerbate impaired growth in children, a condition known as stunting.^^7^^ Characterized by low body weight, short stature, and impaired brain development, stunting can also increase a child’s risk of dying from illnesses such as diarrheal diseases, malaria, and measles. Aflatoxin control is now a priority for the World Bank and other aid groups that count reducing child morbidity and mortality among their major goals.

Second, says John Bowman, a senior agricultural advisor with the U.S. Agency for International Development (USAID), development experts worry that aflatoxins undermine efforts to base food aid on local agriculture—a cheaper, more sustainable approach to aid that both avoids flooding developing country markets with imported grain and breaks dependencies on foreign imports. Barbara Stinson, a senior partner with the nonprofit Meridian Institute, recounts how African stakeholders launched the Partnership for Aflatoxin Control in Africa (PACA) in 2011 after it was discovered that grain shipments headed for distribution in Somalia by the United Nations World Food Programme were contaminated with aflatoxins. That year USAID committed $12 million to PACA’s efforts,^^8^^ and the Bill and Melinda Gates Foundation has endowed a $19.6-million, five-year grant to support the formation and launch of PACA, as well as some pilot studies.^^9^^

Coordinated by the Meridian Institute, PACA’s goal is to make Africa “aflatoxin-safe” using both proven and innovative strategies.^^9^^ According to Stinson, “aflatoxin-safe” means aflatoxin risks should be minimized to the lowest degree possible, with the understanding they can’t be eradicated completely.

Finally, the impact of recent weather extremes suggests that climate change could increase aflatoxin issues worldwide. For instance, much of the corn in the U.S. Corn Belt—including portions of Kansas, Nebraska, Iowa, Missouri, Ohio, and Illinois—was infected with the fungus last year, on the heels of the worst drought in half a century.^^10^^ Hurburgh says aflatoxins routinely contaminate crops in hot states like Texas and Arizona, but it was unusual to see such extensive contamination in the cooler Corn Belt states to the north. “There’s a lot of consensus that weather extremes will become more common worldwide,” he says. “And if that’s the case, then we’re going to be dealing with aflatoxin and similar mycotoxin problems more frequently.”

## Introducing Aflatoxins

It’s unclear why *A. flavus* and *A. parasiticus* produce aflatoxins. Scientists have a number of hypotheses. Aflatoxins may trap the free radicals that plants generate to protect themselves from the fungi. Or the fungi may produce aflatoxins to protect themselves from insects.

According to Gary Payne, a plant pathologist and professor at North Carolina State University, *A. flavus* has an unusual heat tolerance; it thrives in temperatures approaching 100°F and continues to grow even at 118°F, which is much hotter than most other fungi can tolerate.

High temperatures weaken corn and other crops, Payne explains, particularly when they don’t get enough water. This can create fissures in developing grain tissues within which *Aspergillus* takes hold. “Moreover, heat stress goes hand in hand with insect-induced injury, and that also boosts crop vulnerability to *A. flavus* infections,” he says. The infections are triggered by combinations of high heat and moisture, either in the field or in storage. But once they take hold, they can persist even under very dry conditions.

Hurburgh points out that aflatoxin molecules are remarkably stable, able to resist even the industrial fermentation processes used to make corn-based ethanol. Residual aflatoxin contamination can wind up in “distillers grains,” the corn sludge left over from fermentation, which is used as livestock feed and in low-cost pet foods. Aflatoxin outbreaks have killed dogs in the United States, but not human consumers, according to Hurburgh.

U.S. users and exporters sample grain elevators extensively, particularly when they suspect contamination, in order to ensure that levels don’t exceed the domestic standard for aflatoxins of 20 ppb,^^11^^ while companies in developed countries that make cereal and other processed foods are “always sampling” for aflatoxin, Payne says. To further limit the possibility of childhood exposure, aflatoxin contamination in milk (which can result from dairy cattle eating contaminated feed) is tightly regulated in the United States with an action guideline of just 0.5 ppb^^12^^ and in the European Union with an even more stringent maximum level of 0.05 ppb.^^13^^ Hurburgh says that monitoring increases in years when the weather creates more risk.

## A Hidden Risk

By comparison, says Felicia Wu, Hannah Distinguished Professor of Food Science and Human Nutrition at Michigan State University, aflatoxin monitoring is rare in developing countries, with the exception of grains and nuts headed for export. “Even in countries where standards for aflatoxin exist, they may not always be enforced,” she says. “Subsistence farmers just eat what they grow; lack of awareness about aflatoxin is a serious issue.” In some cases, moldy foods are eaten simply because other foods might not be available, says Bowman.

A big part of the problem in Africa, according to Hurburgh, is that farmers do not have mechanical drying equipment; early harvest and rapid mechanical drying are big factors in U.S. farmers’ ability to keep aflatoxin levels down in difficult years. “The toxin forms at 25% moisture, down to about 17%,” Hurburgh says. “If the corn has to stand in the field warm and humid at these moistures, the risk is high.”

Even when they know about aflatoxin, inspectors in the developing world can’t always spot harmful *Aspergillus* infestations with the naked eye. For the most part, aflatoxin monitoring requires expensive technologies, such as ELISA test kits (which cost US$15 or more per sample), high-performance liquid chromatography, and mass spectrometers, which aren’t readily available to governments and marketers in poor countries, Wu says. Moreover, sampling is far from foolproof—a single ear of corn might have just a few highly infected kernels, which makes the contamination hard to detect. “Blended into a load of grain, you won’t find those kernels unless you sample in the right place,” explains Payne.

Francesca Nelson, a Kenya-based food security and nutrition advisor with USAID, says a variety of low-cost aflatoxin test kits for use in poor countries are now under development. But the best way to minimize aflatoxin risk, she emphasizes, is to try to prevent the poison from getting into foods in the first place. “Once it’s in the food supply, aflatoxin becomes very difficult to manage,” she says. “It tends to affect crops across very large agricultural areas.”

## Liver Effects

Aflatoxins were first recognized as a health risk in the mid-twentieth century, when they were revealed as the cause of death among turkeys eating *A. flavus–*contaminated feed.^^14^^ Studies later showed that aflatoxin B_1_ causes liver cancer in nonhuman primates, rodents, and fish,^^15^^^^,^^^^16^^ as well as in humans. The International Agency for Research on Cancer (IARC) of the World Health Organization classified aflatoxins as a Group 1 carcinogen (definitely carcinogenic to humans) in 1987, and analysis of additional data in 2002 reaffirmed this categorization.^^17^^

Liver failure also occurs in highly exposed humans suffering from a condition known as aflatoxicosis. During an outbreak of aflatoxicosis that killed over 125 people in Kenya in 2004, victims experienced abdominal pain, pulmonary edema, liver necrosis, and finally death after ingesting doses of aflatoxin B_1_ estimated at 50 mg per day.^^18^^

Mechanistically, P450 enzymes in the liver metabolize aflatoxins to reactive oxygen species that bind with DNA.^^19^^ “There’s a wide range of sensitivity to aflatoxin carcinogenicity across species,” says Thomas Kensler, a professor of pharmacology and chemical biology at the University of Pittsburgh. “Mice are quite resistant relative to trout, which are the most sensitive species. And the big question is where humans fit into that equation—we think somewhere in the middle.”

Epidemiological studies from Qidong and Fusui provinces in China support the carcinogenicity of aflatoxin B_1_ in humans. Both hot spots for liver cancer, these regions relied for years on corn as a staple food. The corn was frequently contaminated with *A. flavus*, Kensler explains, and when economic reform allowed residents of Qidong to shift to imported rice as a staple during the 1980s, the rates of liver cancer began to plummet.^^20^^^^,^^^^21^^ (Kensler adds that the local corn was subsequently used as animal feed. However, he adds, “Most Chinese don’t drink dairy products [because of lactose intolerance], so I don’t think the switch from maize to rice introduced a new exposure paradigm.”)

Wu says that people with hepatitis B face up to a roughly 30-fold elevated cancer risk from aflatoxin, compared with people exposed to aflatoxin only. According to Wu, aflatoxin exposure and hepatitis B are both endemic in the developing world, where prevalence rates for liver cancer range from 16 to 32 times higher than in developed countries.^^22^^

## Evidence for Stunting

Supported by 50 years of data and knowledge of clearly defined biological mechanisms, the evidence on cancer is far more robust than that linking aflatoxins with childhood stunting. Some scientists—notably Kensler and his colleague John Groopman, a professor of environmental health at the Johns Hopkins Bloomberg School of Public Health—emphasize that, although provocative, the data on stunting so far remain inconclusive, largely because of mechanistic data gaps.

“There’s still a reasonable degree of uncertainty over aflatoxins’ role in stunting,” Kensler says. “The evidence is interesting, and it warrants further investigation both in humans and in animals.”

Kitty Cardwell, a National Program leader and plant pathologist with the U.S. Department of Agriculture (USDA), acknowledges that scientists can only speculate on how aflatoxins might induce stunting—they might suppress the immune system, she says, or produce enteric damage that limits the absorption of nutrients. Or stunting could be a result of simple liver toxicity. “No one knows the mechanism,” she says, “but the association is highly significant.”

Cardwell first noticed a relationship between aflatoxin exposure and stunting during the mid-1990s, when she was working for the International Institute of Tropical Agriculture (IITA) in Benin and Nigeria. She and her colleagues had tested nearly 1,000 corn samples from the region and found high aflatoxin B_1_ concentrations in some regions during certain seasons.^^23^^^^,^^^^24^^ Cardwell knew that laboratory data had already associated aflatoxins with faltering growth in weaning animals, and she wondered about the risks to African children, who are often weaned onto corn-based foods.

She measured blood aflatoxin in more than 700 local children and compared the levels with a wide range of developmental end points. And what “leapt off the page,” she says, was a powerful correlation between aflatoxins in blood and impaired growth. “The higher the aflatoxin levels, the lower the growth rates,” she says.

Published in 2002 with a followup paper two years later, these were the first data to show an association between aflatoxins and stunting in human children.^^25^^^^,^^^^26^^ Since then, studies in Togo, Gambia, Ghana, Iran, Kenya, and the United Arab Emirates have shown similar findings, outlined in a review published by Wu and colleagues in 2011.^^18^^

Stinson says research investments still need to be made on aflatoxin exposure and childhood stunting, which in 2010 was estimated to afflict more than 171 million children worldwide.^^27^^ The Bill and Melinda Gates Foundation is planning for additional studies in this area, she says, as are other organizations. Meanwhile, the current evidence on stunting, combined with well-documented risks for liver cancer and aflatoxicosis, justify global efforts to minimize human exposure, she says.

## Confronting the Problem

According to Stinson, reducing those exposures can be accomplished by combining a number of good practices, such as early harvesting during weather patterns that favor *Aspergillus* infections (followed by rapid drying and storage of the crop), separating poor- and good-quality grain, drying to reduce grain moisture content, and using storage containers that minimize temperature and moisture conditions that favor fungal growth. In addition, she says, it helps if farmers and others use good sampling practices and inexpensive tests for aflatoxins to remove bad grain at various points in the agricultural supply chain. In a 2011 publication Wu and colleagues assessed the costs and efficacy associated with a number of other risk-mitigation practices, among them improved irrigation, grain ozonation (which kills aflatoxin B_1_ but also can result in degradation of essential nutrients), and hepatitis B vaccination to lessen liver cancer risks among aflatoxin-exposed individuals.^^28^^

PACA, USAID, and other organizations emphasize promoting field-based biocontrol methods that can prevent *Aspergillus* from taking hold. The leading biocontrol method was developed by Peter Cotty, a research plant pathologist at the USDA and an adjunct professor at the University of Arizona. During the late 1980s Cotty was studying the virulence of different *A. flavus* strains, which number in the thousands. Virulence in this case refers to the strain’s ability to colonize and infect plant seeds. What Cotty found was that atoxigenic strains—meaning those that don’t produce aflatoxins—are as virulent as those strains that do.

Intrigued with that finding, Cotty proposed a novel idea: By inoculating agricultural fields with atoxigenic strains of *A. flavus* at early stages in the crop growing cycle, it might be possible to ward off aflatoxin contamination. Since the atoxigenic strains are equally virulent, he reasoned, deliberate inoculation might give them a growth advantage and a chance to outcompete their toxic counterparts.

Critics found his idea preposterous. In a 1993 article, the *Wall Street Journal* reported that seed companies were “aghast that the government would consider releasing a fungus that would still infect plants even if it doesn’t taint the crop with a carcinogen.”^^29^^ But according to Cotty, other options hadn’t panned out. Scientists have never successfully developed an acceptable fungicide, nor have they been able to breed viable varieties of resistant corn.

Today, Cotty’s biocontrol method, now patented by the USDA Agricultural Research Service, is gaining in popularity. Known as AF36, it’s manufactured and distributed in the southwestern United States by the Arizona Cotton Research and Protection Council for use on corn, cotton, and pistachios. An analogous product is sold in Africa under the name Aflasafe. Syngenta, a global agribusiness firm, manufactures and markets a separate product known as Afla-guard® for U.S. distribution and use on corn and peanuts.

Cotty also collaborates with Ranajit Bandyopadhyay, a plant pathologist with the IITA, who is leading efforts to develop atoxigenic strains for use in Africa. According to Bandyopadhyay, those strains come from the African countries in which they’re applied, which is important because it means they’re already adapted to the natural environment and thus more likely to colonize targeted crop plants.

## Building Biocontrols

To develop country-specific Aflasafe products, Bandyopadhyay and Cotty start with a collection of 5,000 strains obtained from widely distributed crop samples in each country. They use a series of selection criteria to narrow the number to roughly a dozen nontoxic strains, each screened for genetic stability, the ability to colonize target crops, and persistence in the field. Screening also ensures the strains have defects in one or more genes associated with aflatoxin production, Bandyopadhyay says. Ultimately they select four local atoxigenic strains to go in each customized product.

Citing evidence gathered during IITA field studies in Nigeria, Bandyopadhyay claims the biocontrol method can reduce aflatoxin contamination in corn and groundnuts by 80–90%, in some cases even as much as 99%.^^30^^ He adds that IITA is now working with a variety of partners to promote biocontrol in Nigeria, Senegal, Ghana, Kenya, Tanzania, Mozambique, Zambia, and Burkina Faso, with plans to expand into several East African countries over the coming three years. Likewise, the USDA and IITA have a pilot program investigating Aflasafe uses in Kenya that’s been running for nearly three years, according to Nelson.

But the main challenge, Bandyopadhyay says, is convincing local farmers that biocontrol is worth the investment. “How do you sell something that has a hidden benefit?” he asks. “The problem with aflatoxin is that you can’t see it. And unless the levels are very high, the expression of health impacts isn’t immediately obvious. For instance, it can take decades to develop cancer.” Still during an unpublished willingness-to-pay study conducted in Nigeria, Bandyopadhyay says IITA researchers found that when informed of aflatoxins’ health risks and the economic benefits of control, 82% of farmers queried said they’d pay US$12–15 per hectare for biocontrol treatments.

Three years ago, a World Bank–funded agriculture development project purchased 8 tons of Aflasafe for distribution in Nigeria and covered 50% of the out-of-pocket cost for farmers who purchased it. According to Bandyopadhyay, the project lowered that subsidy to 25% the next year and eliminated it completely in 2013. Meanwhile, aid groups and governments are experimenting with other incentives, such as bundling Aflasafe into technical packages that also include better seed, fertilizer, and pest controls. “That’s one way to jumpstart economic demand,” explains Cardwell.

Now, under a subcontract to the Meridian Institute, IITA is building a demonstration-scale manufacturing facility on its own campus that will produce 5 tons of Aflasafe an hour, enough to cover 4,000 hectares a day. According to Bandyopadhyay, the overall goal of that effort is to enable manufacturers to produce enough Aflasafe to treat a million hectares in Africa within the next few years and reduce aflatoxin contamination in those areas by 90%.

## Next Steps

Meanwhile, experts are still trying to breed aflatoxin-resistant varieties of corn and other crops. Despite years of effort, they have yet to produce a single commercially viable cultivar. But Robert Brown, a research plant pathologist with the Agricultural Research Service, says he and his collaborators have bred several potentially viable varieties from aflatoxin-resistant samples collected in the United States and Africa. According to Brown’s unpublished data, aflatoxin levels are nearly 87-fold lower in the resistant varieties than in nonresistant corn kernels.

USAID’s Bowman says that along with biocontrol, resistance breeding remains an important part of strategies to minimize aflatoxin exposure in the developing world. The trick, Payne says, is to breed resistant lines with other commercially viable attributes, such as high yield.

The lack of consistent standards within African countries and elsewhere in the world is also problematic, Nelson adds. PACA and other groups are working to support development of harmonized standards in Africa, but selecting a shared value is a complex and arduous prospect. “Countries don’t want to be held to a standard they don’t think they can achieve,” Nelson explains.

Still, Stinson says, aflatoxins are a major trade issue because some 15 African countries have aflatoxin limits above which crops are not allowed into the country. “Individual countries need to establish standards, train farmers, [and] work through agricultural extensions to control aflatoxin,” she says. “Each country with an aflatoxin problem needs an aflatoxin action plan.”

Ultimately, tackling aflatoxins in the developing world will require a profound social transformation, Cardwell emphasizes. “We need a system that makes aflatoxin control worthwhile,” she says. “Technically, it’s a no-brainer, but from a social and development context it’s extremely complex.”

## References

[1] Njoroge E. Kenya to mop up contaminated maize. Capital News [Nairobi, Kenya], News section, online edition (10 May 2010). Available: http://goo.gl/yyQ69O [accessed 26 August 2013]

[2] Probst C Identification of atoxigenic *Aspergillus flavus* isolates to reduce aflatoxin contamination of maize in Kenya. Plant Dis 95 2 212 218 2011; http//dx..org/10.1094/PDIS-06-10-0438 30743416

[3] CDC. National Health and Nutrition Examination Survey. 1999–2000 Data Documentation, Codebook, and Frequencies. Aflatoxin B_1_-Lysine Concentration in Serum (SSAFB_A). Data File: SSAFB_A.xpt. Atlanta, GA:U.S. Centers for Disease Control and Prevention (August 2012). Available: http://goo.gl/XeEtZj [accessed 26 August 2013]

[4] Yard EE Human aflatoxin exposure in Kenya, 2007: a cross-sectional study. Food Addit Contam Part A Chem Anal Control Expo Risk Assess 30 7 1322 1331 2013; http//dx..org/10.1080/19440049.2013.789558 23767939 PMC3725670

[5] CDC. Aflatoxin [website]. Atlanta, GA:U.S. Centers for Disease Control and Prevention (updated 13 January 2013). Available: http://www.cdc.gov/nceh/hsb/chemicals/aflatoxin.htm [accessed 26 August 2013]

[6] Liu Y Wu F. Global burden of aflatoxin-induced hepatocellular carcinoma: a risk assessment. Environ Health Perspect 118 6 818 824 2010; http//dx..org/10.1289/ehp.0901388 20172840 PMC2898859

[7] Khlangwiset P Aflatoxins and growth impairment: a review. Crit Rev Toxicol 41 9 740 755 2011; http//dx..org/10.3109/10408444.2011.575766 21711088

[8] USAID. U.S. Announces Support for the Africa-Led Partnership for Aflatoxin Control in Africa [press release]. Washington, DC:U.S. Agency for International Development (10 June 2011). Available: http://goo.gl/XEDBsP [accessed 26 August 2013]

[9] Meridian Institute. Support for Innovative Partnership for Aflatoxin Control in Africa Funded by Bill & Melinda Gates Foundation and Department for International Development of the United Kingdom of Great Britain and Northern Ireland (DfID) [press release]. Washington, DC:Meridian Institute (23 February 2012). Available: http://www.aflatoxinpartnership.org/Press_Release.aspx [accessed 26 August 2013]

[10] Ingwersen J. US grain sector on high alert for aflatoxin in drought-hit corn. Reuters, online edition (30 August 2012). Available: http://goo.gl/mBHRlf [accessed 26 August 2013]

[11] FDA. Compliance Manuals. Compliance Policy Guides. Section 555.400. Foods—Adulteration with Aflatoxin. Silver Spring, MD:U.S. Food and Drug Administration, U.S. Department of Health and Human Services (updated 9 December 2009). Available: http://goo.gl/xq16nd [accessed 26 August 2013]

[12] FDA. Compliance Manuals. Compliance Policy Guides. Section 527.400. Whole Milk, Lowfat Milk, Skim Milk—Aflatoxin M_1_ Silver Spring, MD:U.S. Food and Drug Administration, U.S. Department of Health and Human Services (updated 12 August 2009). Available: http://goo.gl/e48EA7 [accessed 26 August 2013]

[13] Commission Regulation (EC) No. 1881/2006, OJ L 364 of 20 December 2006. Available: http://goo.gl/IMiS9I [accessed 26 August 2013]

[14] Kensler TW Aflatoxin: a 50-year odyssey of mechanistic and translational toxicology. Toxicol Sci 120 suppl1 S28 S48 2011; http//dx..org/10.1093/toxsci/kfq283 20881231 PMC3043084

[15] Busby WFJ, Wogan GN. Aflatoxins. In: Chemical Carcinogens (Searle CD, ed.). Washington, DC:American Chemical Society (1984)

[16] Eaton DL, Groopman JD. The Toxicology of Aflatoxins: Human Health, Veterinary, and Agricultural Significance. San Diego, CA:Academic Press, Inc. (1994)

[17] IARC. Some Traditional Herbal Medicines, Some Mycotoxins, Naphthalene and Styrene, Volume 82. Summary of Data Reported and Evaluation. Lyon, France:International Agency for Research on Cancer, World Health Organization (updated 4 December 2002). Available: http://goo.gl/PTFElR [accessed 26 August 2013]

[18] Probst C Outbreak of an acute aflatoxicosis in Kenya in 2004: identification of the causal agent. Appl Environ Microbiol 73 8 2762 2764 2007; http//dx..org/10.1128/AEM.02370-06 17308181 PMC1855601

[19] Khlangwiset P Aflatoxins and growth impairment: a review. Crit Rev Toxicol 41 9 740 755 2011; http//dx..org/10.3109/10408444.2011.575766 21711088

[20] Chen JG Reduced aflatoxin exposure presages decline in liver cancer mortality in an endemic region in China. Cancer Prev Res; http//dx..org/10.1158/1940-6207.CAPR-13-0168[online 20 August 2013] 10.1158/1940-6207.CAPR-13-0168 PMC3800239 23963804

[21] Chen JG Trends in the mortality of liver cancer in Qidong, China: an analysis of fifty years [in Chinese] Zhonghua Zhong Liu Za Zhi 34 7 532 537 2012; http://www.ncbi.nlm.nih.gov/pubmed/22967473 22967473 10.3760/cma.j.issn.0253-3766.2012.07.012

[22] Liu Y Wu F. Global burden of aflatoxin-induced hepatocellular carcinoma: a risk assessment. Environ Health Perspect 118 6 818 824 2010; http//dx..org/10.1289/ehp.0901388 20172840 PMC2898859

[23] Hell K The influence of storage practices on aflatoxin contamination in maize in four agroecological zones of Benin, West Africa. J Stored Prod Res 36 4 365 382 2000; http//dx..org/10.1016/S0022-474X(99)00056-9 10880814

[24] Udoh JM Storage structures and aflatoxin content of maize in five agroecological zones of Nigeria. J Stored Prod Res 36 2 187 201 2000; http//dx..org/10.1016/S0022-474X(99)00042-9

[25] Gong YY Dietary aflatoxin exposure and impaired growth in young children from Benin and Togo: cross sectional study. BMJ 325 7354 20 21 2002; http//dx..org/10.1136/bmj.325.7354.20 12098724 PMC116667

[26] Gong Y Postweaning exposure to aflatoxin results in impaired child growth: a longitudinal study in Benin, West Africa. Environ Health Perspect 112 13 1334 1338 2004; http//dx..org/10.1289/ehp.6954 15345349 PMC1247526

[27] de Onis M Prevalence and trends of stunting among pre-school children 1990–2020. Public Health Nutr 15 1 142 148 2011; http//dx..org/10.1017/S1368980011001315 21752311

[28] Khlangwiset P Wu F. Costs and efficacy of public health interventions to reduce aflatoxin-induced human disease. Food Addit Contam Part A Chem Anal Control Expo Risk Assess 27 7 998 1014 2010; http//dx..org/10.1080/19440041003677475 20419532 PMC2885555

[29] Kilman S. Food-safety strategy pits germ against germ. The Wall Street Journal (New York, NY), B2 (16 March 1993)

[30] IITA. Annual Report 2011. Croydon, UK:International Institute of Tropical Agriculture (2011). Available: http://goo.gl/Oax5fI [accessed 26 August 2013]

