# Erratum: “Breaking the Mold: New Strategies for Fighting Aflatoxins”

**DOI:** 10.1289/ehp.122-A148

**Published:** 2014-06-01

The Focus article “Breaking the Mold: New Strategies for Fighting Aflatoxins” [Environ Health Perspect 121:A270–A275 (2013); http://dx.doi.org/10.1289/ehp.121-A270] incorrectly identified Qidong and Fusui as provinces in China. Qidong is a city, and Fusui is a county.

*EHP* regrets the error.

